# Medical students and controversial ethical issues: results from the multicenter study SBRAME

**DOI:** 10.1186/1472-6939-15-85

**Published:** 2014-12-15

**Authors:** Giancarlo Lucchetti, Leandro Romani de Oliveira, José Roberto Leite, Alessandra Lamas Granero Lucchetti, for the SBRAME Collaborators

**Affiliations:** Department of Medicine, Federal University of Juiz de Fora, Juiz de Fora, Brazil; Department of Psychobiology, Federal University of São Paulo, São Paulo, Brazil; Rua Dona Elisa 150 apto. 153B, Barra Funda, São Paulo SP, 01155-030 Brazil

**Keywords:** Ethics, Religious ethics, Abortion, Euthanasia, Medical education

## Abstract

**Background:**

Medical students(MS) will face ethical issues throughout their lives as doctors. The present study aims to investigate medical students’ opinions on controversial ethical issues and factors associated with these opinions.

**Methods:**

SBRAME (Spirituality and Brazilian Medical Education) is a multicenter study involving 12 Brazilian medical schools with 5950 MS. Participants completed a questionnaire that collected information on socio-demographic data, medical schools characteristics, religious beliefs and opinions on controversial ethical issues. Of all MS, 3630 participated in the survey (61.0%).

**Results:**

The sample was 53.8% women and the mean age was 22.5 years. In general, most MS have no objections to prescription of birth control (90.8%), adult stem cell use (87.5%), embryonic stem cell use (82.0%) and abortion for genetic reasons (51.2%). Approximately half of students have no objections to human cloning (47.3%), 45.7% to withdrawal of artificial life support, 41.4% to euthanasia and 23.3% to abortion for failed contraception. Socio-demographic data such as age, gender and income had little influence on MS opinions. On the other hand, medical schools characteristics (number of medical students in the university, year of medical school foundation, location of the university and type of university) and religious aspects (religious affiliation, religious attendance, non-organizational religiousness and intrinsic religiousness) were highly correlated with their opinions. In general, MS with more supportive opinions on controversial ethical issues were less religious and from non-traditional (newer), urban, public and bigger universities.

**Conclusion:**

The current study reveals MS have different opinions regarding controversial ethical issues. Noteworthy, these opinions seem to be shaped more by university characteristics and religious beliefs than socio-demographic data.

## Background

“Ethics is an academic discipline that reflects critically upon values and meaning of human experience, considers ways to mediate differences in values through moral argument and examines the right or wrong of human acts”. This definition, taken from an ethics book [1], reflects the importance of ethics education to medical students (MS) formation.

According to Miles et al. [2], medical ethics deals with the examination of the role of values in the doctor’s relationship with patients, colleagues and other providers, and society. Given its importance in undergraduate medical education, most medical schools worldwide have courses dealing with this issue [3].

In fact, MS will face ethical issues throughout their lives as doctors. Feudtner et al. [4] evaluated 665 MS and found that 58% reported having done something they believed was unethical, 98% had heard physicians refer derogatorily to patients; 61% had witnessed what they believed to be unethical behavior by other medical team members, and of these students, 54% felt like accomplices.

MS will also come up against controversial ethical issues (medical practices), about which they may have moral qualms [5]. Curlin et al. [5] have investigated US physicians’ opinions about controversial clinical practices and found 52% objected abortion due to failed contraception, 42% objected prescription of birth control to adolescents without parental consent and 17% objected terminal sedation. In addition, most physicians believed that it is ethically permissible for doctors to explain their moral objections to patients (63%) and to refer the patient to another clinician who does not object to the requested procedure (71%).

Euthanasia, legal abortion and termination of life-sustaining treatment are some examples of ethical issues that MS and doctors should have knowledge and be trained to deal with it.

Among several factors which could have an influence on doctors and MS ethical opinions; religious/cultural issues [6], undergraduate training [7] and medical school characteristics [8] seem to play important roles.

Religious beliefs are associated with differing attitudes in the clinical encounter [6] and differing attitudes towards several of the ethical controversies of assisted reproductive technologies and legal abortion [9]. Studies have shown religious physicians are less sympathetic to euthanasia and physician-assisted suicide [10] and are more likely to report that doctors may describe their objections to patients [5].

Undergraduate training is responsible for promoting a significant increase in the growth and development of moral reasoning in medical students [7] and should offer students the tools to analyze ethical issues and to formulate a cogent and well-defended position without, however, dictate a particular substantive outcome [11].

Finally, since there are important differences between medical schools characteristics [8] such as size, religious affiliation, curriculum, syllabi, period of teaching, tradition and location, these characteristics may impact MS ethical opinions.

Hence, understanding MS opinions and what shapes their ethical views could help to provide further evidence to this field and to create new teaching strategies. Therefore, the present study aims to investigate medical students’ opinions on controversial ethical issues and factors associated with these opinions.

## Methods

### Study design

SBRAME (Spirituality and Brazilian Medical Education) is a cross-sectional, multicenter study involving 12 Brazilian medical schools that enrolled 5950 medical students (MS) [12]. The study was carried out from June 2010 to September 2011 and was coordinated by the Universidade Federal de São Paulo (UNIFESP), Universidade Federal de Juiz de Fora and Brazilian Medical Spiritist Association, Brazil. A previous article has been already published with this database aiming to evaluate the relationship between spirituality/religiosity and the attitudes, beliefs and experiences of medical students in Brazil [13]. The present study, on the other hand, focus on controversial ethical issues and associated factors.

### Participating institutions

The following medical schools participated in the study: Universidade Federal de São Paulo (UNIFESP) – São Paulo (570 students) – Public (Federal); Faculdade de Medicina de Marília (FAMEMA) – Marília (460 students) – Public (State); Centro Universitário Lusíadas (Lusíadas) – Santos (460 students) – Private; Faculdade de Medicina de São José do Rio Preto (FAMERP) – São José do Rio Preto (370 students) – Public (State); Pontifícia Universidade Católica de Sorocaba (PUC-SP) – Sorocaba (580 students) – Private; Universidade Metropolitana de Santos (UNIMES) – Santos (480 students) – Private; Universidade Nove de Julho (UNINOVE) – São Paulo (580 students) – Private; Faculdade de Ciências Médicas da Santa Casa de São Paulo (Santa Casa) – São Paulo (570 students) – Private; Faculdade de Medicina do ABC (FMABC) – São Paulo (580 students) – Private; Universidade Federal de Santa Maria (UFSM) – Santa Maria (580 students) – Public (Federal); Universidade Federal do Mato Grosso do Sul (UFMS) – Cuiabá (360 students) – Public (Federal) and Faculdade de Medicina de Jundiaí (360 students) – Private.

### Training of researchers

First, a meeting (with at least one member of each medical school) was held to discuss the objectives and assess each institution’s willingness to participate in this study. Next, research supervisors and interviewers from each medical school were trained using a common manual and supplemented by web-based training.

### Procedures and participants’ selection

All MS officially registered in the 12 medical schools were invited by the researchers to take part in the study. MS were personally approached before or after classes and during breaks.

### Data collection instrument

Participants completed a self-administered, multiple choice 43-item questionnaire, which was adapted and expanded from other pilot studies carried out in Brazil [14–16] and collected the following information:

- Socio-demographic data: gender, age, family income, ethnicity, religious affiliation, undergraduate year and desired medical specialty.
- Controversial ethical issues: MS were asked about controversial issues in medicine using an instrument adapted from Curlin et al. [5, 17] in previous studies. The following statement was used: “Please note if you object to any of the following medical practices, and if so, whether your objection is for religious reasons, reasons unrelated to religion, or both”. The issues were: Euthanasia, Withdrawal of artificial life support, Abortion for congenital abnormalities, Abortion for failed contraception, Prescription of birth control, embryonic stem cell use, adult stem cell use and human cloning. Possible answers were: “I have no objection”, “I have religious objections”, “I have non-religious objections”, “I have religious and no-religious objections” and “I have no opinion”.
- Medical School characteristics: Number of medical students, type of university (public or private), year of medical School foundation and location of the university (urban or rural).
- Religiosity and beliefs: For assessing the religious aspects of participants, the Duke Religious Index (DUREL) validated into Portuguese was used [18]. DUREL is a five-item measure of religious involvement made up of three subscales: (1) organizational religious behavior - religious attendance (1 item), (2) non-organizational religious behavior - praying, scripture reading, meditation, among others (1 item), and (3) intrinsic religious motivation (3 items). Response options are on a 5- or 6-point Likert scale. We have also asked whether the MS believes in life after death and in soul/spirit with possible answers “yes”, “no” and “I have no opinion”.
- Happiness (adapted from Curlin et al. [5]) and Satisfaction with the medical course: assessed through the questions: (1) “If you were to consider your life in general these days, how happy or unhappy would you say you are, on the whole?” with possible answers “Very happy”, “Happy”, “Not very happy” and “Not at all happy”; (2) “How much are you satisfied studying to become a doctor?” “Very satisfied”, “Satisfied”, “Not very satisfied” and “Not at all satisfied”.

### Statistical analysis

Data were entered into an Excel database and analyzed using Statistical Package for Social Sciences program (SPSS), version 17.0 (SPSS Inc). Descriptive statistics were used to describe the range of responses. For categorical variables, the descriptive statistics are reported as numbers and percentages. For continuous data, the descriptive statistics include mean and standard deviations.

For assessing factors associated with MS’ opinions on each ethical issue, a logistic regression was used with the following variables:

- Dependent variables (1 = no objections; 0 = with objections): euthanasia, withdrawal of artificial life support, abortion for congenital abnormalities, abortion for failed contraception, prescription of birth control, embryonic stem cell use, adult stem cell use and human cloning.
- Independent variables: gender, age, undergraduate year, Number of medical students in the university, type of university (public or private), Year of medical School foundation, income, location of the university (urban or rural), intrinsic religiousness, religiousness attendance, non-organizational religiousness, happiness (“Very happy” to “Not at all happy”), satisfaction with the course (“Very satisfied” to ”Not at all satisfied”), believe in life after death (Yes or no), believe in soul (Yes or no), religious affiliation and desired medical specialty (Internal medicine, gynecology/obstetrics, pediatrics, surgery and others).

All independent variables were included in the analysis, and only those identified by the foreward logistic regression model as independently associated with the dependent variable were included in the model. Goodness of fit was evaluated by the Hosmer-Lemeshow test and Nagelkerke R square. All confidence intervals are 95% and p < 0.05 was considered significant.

### Ethical issues

Participants gave written informed consent and the study was approved by the ethics committee of Universidade Federal de São Paulo and all other medical school committees.

## Results

### Sample

A total of 3630 medical students participated in the study with a response rate of 61.0%. The most common reasons for not participating were: refused, did not wish to sign the consent form, were interviewers in the study, or were absent at the time of the survey.

### Socio-demographic characteristics

**Table 1:** **Socio-demographic and religious characteristics of the sample**

Socio-demographic and religious characteristics	n	%
Gender		
Male	1657	46.2
Female	1931	53.8
Family income		
Less than	863	24.3
R$4746 (US$2109) to R$ 8136 (US$ 3616)	916	25.8
More than RS 8136 (US$ 3616)	1775	48.9
Race		
White	2979	82.2
Mixed	128	3.4
Black	43	1.2
Asian	356	9.8
Other	119	3.3
Age (Mean, SD)	22.5	(4.6)
Religious affiliation		
No religion and not believe in God	300	8.3
No religion, but believe in God	923	25.6
Catholics	1245	34.6
Protestant Evangelicals	291	8.0
Spiritists	470	13.0
Others	370	10.5
How often do you attend church or other religious meetings?		
Once a week or more	649	18.2
Less than once a week and more than once a year	1628	45.2
Once a year or less/Never	1320	36.6
How often do you spend time in private religious activities (prayer, Bible study, etc.?)		
Daily or more	1173	32.7
Less than daily and at least once a week	829	23.1
Less than once a week/never	1597	44.2
Do you believe that after death, the soul/spirit remains alive?		
Yes	2395	66.8
No	520	14.5
No opinion	669	18.7
Do you believe the human body is composed by a body and a soul?		
Yes	2841	78.9
No	336	9.3
No opinion	421	11.8

Most MS were women (53.8%), mean age was 22.5 years (SD: 4.6), 82.2% were white, and 48.9% had an average family income of more than US$3616 per month. Participants were equally distributed in the medical school years: 612 (16.9%) in the first year; 752 (20.7%) in the second year; 559 (15.4%) in the third year; 635 (17.5%) in the fourth year; 579 (15.9%) in the fifth year, and 485 (13.6%) in the sixth year (Table 1).

### Institutions’ characteristics

There were 7 (58.3%) private schools and 5 (41.7%) public schools (3 federal and 2 state), most institutions (83.3%) were from South-east Brazil (most wealthy region in Brazil) and 5 (41.7%) were from urban centers. The mean year of medical school foundation was 1967.0 (SD: 18.6) varying from 1933 to 2003 and the number of students per medical school was approximately 479.1 (SD: 94.3) varying from 360 to 580. All medical schools have a course on medical ethics.

### Medical students’ spiritual and religious beliefs

Most MS (66.1%) had a religious affiliation (Catholics followed by Evangelicals); believed in God (84.2%); attended religious services less than once a week (81.8%); spend more than once a week in private religious activities (55.8%) and believe that human body included a soul (78.9%). The mean of DUREL intrinsic religiosity was 9.63 (SD: 3.69), which ranged from 3 (low intrinsic religiosity) to 15 (high intrinsic religiosity).

### Controversial ethical issues

Table 2 presents MS opinions on controversial ethical issues. In general, most MS have no objections to prescription of birth control (90.8%), adult stem cell use (87.5%), embryonic stem cell use (82.0%) and abortion for genetic reasons (51.2%). Approximately half of students have no objections to human cloning (47.3%), 45.7% to withdrawal of artificial life support, 41.4% to euthanasia and 23.3% to abortion for failed contraception. Both religious and non religious issues were the most common reasons for objecting some ethical issues.

**Table 2:** **Medical Students opinions on controversial ethical issues**

Ethical issue	I have no objections	I have religious objections	I have non-religious objections	I have religious and non-religious objections	I have no opinion
Euthanasia	1468 (41.4%)	259 (7.3%)	531 (15.1%)	653 (18.4%)	631 (17.8%)
Life support withdrawal	1621 (45.7%)	185 (5.2%)	665 (18.7%)	532 (15.0%)	547 (15.4%)
Abortion for Genetic Reasons	1818 (51.2%)	294 (8.3%)	487 (13.7%)	570 (16.2%)	382 (10.8%)
Abortion from failed contraception	829 (23.3%)	304 (8.6%)	1076 (30.3%)	1076 (30.3%)	270 (7.6%)
Prescription contraceptives	3216 (90.8%)	44 (1.2%)	142 (4.1%)	36 (1.0%)	102 (2.9%)
Embryonic stem cell use	2908 (82.0%)	94 (2.6%)	162 (4.6%)	121 (3.4%)	263 (7.4%)
Adult stem cell use	3106 (87.5%)	44 (1.2%)	125 (3.5%)	71 (2.0%)	205 (5.8%)
Human cloning	1678 (47.3%)	166 (4.7%)	720 (20.3%)	502 (14.1%)	484 (13.6%)

### Factors associated with MS opinions on controversial ethical issues

Tables 3 and 4 present the factors related to MS opinions on controversial ethical issues. Socio-demographic data such as age, gender and income had little influence on MS opinions. On the other hand, medical schools characteristics (number of medical students in the university, year of medical school foundation, location of the university and type of university) and religious aspects (religious affiliation, religious attendance, non-organizational religiousness and intrinsic religiousness) were highly correlated with their opinions. In general, MS with more supportive opinions on controversial ethical issues were less religious and from non-traditional (newer), urban, public and bigger universities.

**Table 3:** **Medical students opinions on Euthanasia, withdrawal of artificial life support, abortion for congenital abnormalities and abortion for failed contraception**

Ethical issue	OR	95% CI	p
**Euthanasia** ^**a**^			
Number of medical students in the university	1.002	1.000-1.003	0.005
Year of medical School foundation	1.007	1.001-1.013	0.029
Intrinsic religiousness	0.922	0.886-0.959	<0.001
Religious attendance	0.678	0.616-0.746	<0.001
Believe in soul	0.573	0.383-0.858	0.007
Desired Medical specialty			
Internal medicine	1.000		
Gynecology/obstetrics	0.794	0.523-1.205	0.278
Pediatrics	0.635	0.443-0.909	0.013
Surgery	1.065	0.817-1.388	0.641
Other	1.068	0.825-1.381	0.619
Religious affiliation			
No affiliation	1.000		
Evangelical	0.791	0.508-1.232	0.300
Catholics	1.063	0.808-1.398	0.661
Spiritist	0.533	0.381-0.746	<0.001
Other	1.132	0.778-1.649	0.517
**Withdrawal of artificial life support** ^**b**^			
Gender (Female)	0.819	0.681-0.986	0.035
Location of the university (urban)	1.308	1.090-1.571	0.004
Income	1.111	1.019-1.211	0.017
Intrinsic religiousness	0.936	0.905-0.968	<0.001
Religious attendance	0.733	0.673-0.798	<0.001
**Abortion for congenital abnormalities** ^**c**^			
Age	1.039	1.009-1.069	0.009
Year of medical School foundation	1.009	1.003-1,014	0.003
Intrinsic religiousness	0.918	0.883-0.954	<0.001
Religious attendance	0.725	0.660-0.797	<0.001
Non-organizational religiousness	0.921	0.858-0.989	0.024
Desired Medical specialty			
Internal medicine	1.000		
Gynecology/obstetrics	1.311	0.885-1.942	0.176
Pediatrics	0.877	0.629-1.222	0.437
Surgery	1.485	1.156-1.906	0.002
Other	1.102	0.866-1.402	0.430
**Abortion for failed contraception** ^**d**^			
Type of university (Public)	0.665	0.535-0.826	<0.001
Intrinsic religiousness	0.909	0.874-0.947	<0.001
Religious attendance	0.804	0.723-0.894	<0.001
Belief in life after death	0.648	0.487-0.864	0.003
Religious affiliation			
No affiliation	1.000		
Evangelical	0.667	0.400-1.113	0.121
Catholics	0.719	0.540-0.957	0.024
Spiritist	0.523	0.354-0.773	0.001
Other	1.159	0.790-1.698	0.451

^a^Hosmer-Lemeshow: 10.427, p = 0.236, Nagelkerke R square = 0.239.

^b^Hosmer-Lemeshow: 5.856, p = 0.663, Nagelkerke R square = 0.130.

^c^Hosmer-Lemeshow: 12.605, p = 0.126, Nagelkerke R square = 0.181.

^d^Hosmer-Lemeshow: 10.308, p = 0.244, Nagelkerke R square = 0.166.

Variables: gender, age, race, undergraduate year, Number of medical students in the university, type of university (public or private), Year of medical School foundation, income, location of the university (urban or rural), intrinsic religiousness, religiousness attendance, non-organizational religiousness, happiness, satisfaction with the course, believe in life after death, believe in soul, religious affiliation and desired medical specialty.

**Table 4:** **Medical students opinions on prescription of birth control, embryonic stem cell use, adult stem cell use and human cloning**

Ethical issue	OR	95% CI	p
**Prescription of birth control** ^**a**^			
Undergraduate year	0.849	0.749-0.963	0.011
Number of medical students in the university	0.993	0.989-0.996	<0.001
Type of university (Public)	6.351	3.809-10.591	<0.001
Year of medical School foundation	1.035	1.020-1.050	<0.001
Location of the university (urban)	6.872	4.254-11.101	<0.001
Religious affiliation			
No affiliation	1.000		
Evangelical	0.976	0.477-1.998	0.947
Catholics	1.448	0.813-2.579	0.209
Spiritist	3.596	1.574-8.212	0.002
Other	1.063	0.532-2.125	0.862
Religious attendance	0.577	0.489-0.681	<0.001
**Embryonic stem cell use** ^**b**^			
Type of university (Public)	1.923	1.412-2.619	<0.001
Year of medical School foundation	1.033	1.023-1.044	<0.001
Location of the university (urban)	1.582	1.193-2.097	0.001
Intrinsic religiousness	0.944	0.891-1.000	0.048
Religious attendance	0.591	0.520-0.672	<0.001
**Adult stem cell use** ^**c**^			
Undergraduate year	0.859	0.771-0.957	0.006
Number of medical students in the university	0.996	0.993-0.999	0.004
Type of university (Public)	4.864	3.155-7.500	<0.001
Year of medical School foundation	1.033	1.019-1.046	<0.001
Location of the university (urban)	4.462	2.931-6.793	<0.001
Religious attendance	0.696	0.622-0.779	<0.001
**Human cloning** ^**d**^			
Gender (Female)	0.711	0.595-0.848	<0.001
Age	1.045	1.013-1.079	0.006
Undergraduate year	0.899	0.847-0.954	<0.001
Religious attendance	0.799	0.750-0.851	<0.001

^a^Hosmer-Lemeshow: 107.812, p = <0.001, Nagelkerke R square = 0.324.

^b^Hosmer-Lemeshow: 20.490, p = 0.009, Nagelkerke R square = 0.180.

^c^Hosmer-Lemeshow: 56.872, p = <0.001, Nagelkerke R square = 0.184.

^d^Hosmer-Lemeshow: 3.563, p = 894, Nagelkerke R square = 0.052.

Variables: gender, age, race, undergraduate year, Number of medical students in the university, type of university (public or private), Year of medical School foundation, income, location of the university (urban or rural), intrinsic religiousness, religiousness attendance, non-organizational religiousness, happiness, satisfaction with the course, believe in life after death, believe in soul, religious affiliation and desired medical specialty.

## Discussion

In relation to other small MS Brazilian studies, we found similar opinions on embryonic stem cell use [19], less supportive opinions on abortion for congenital abnormalities, on withdrawal of artificial life support [20] and more [21] and less [20] supportive opinions on euthanasia. These conflicting results highlight the role of different institutions and cultures on MS opinions.

In general, the current study reveals that MS opinions on controversial ethical issues are related to religious beliefs and medical school characteristics. In fact, we found religious beliefs were strongly associated with MS opinions on these controversial ethical issues. These findings were in accordance with other studies in this field which evaluated physicians. Baume et al. [10] investigated 1238 Australian doctors and found doctors claiming to be agnostic or atheist were more likely to favour and to practise euthanasia and those who identified with any religion were more likely to be opposed. Along the same line, Cohen et al. [22] evaluated physicians in six countries and found religious physicians have less accepting attitudes and less willingness to hasten the patient’s death, Curlin et al. [17] found highly religious physicians are more likely to object to both physician-assisted suicide and terminal sedation, and Aiyer et al. [23] found a strong relationship between physician's decision not to perform abortions and ethical and religious beliefs. All these findings corroborate with our study, in which, MS opinions were influenced by their religious beliefs.

The role of medical school characteristics is less established than religious beliefs. It is well known that medical schools differ in their curriculum, structure and missions [8]. Although all medical schools evaluated in the present study have medical ethics in their curriculum, their teaching methods vary, which could have an influence on MS learning. In a recent study, Billings et al. [24] evaluated 1455 fourth-year medical students at 62 US medical schools and found MS exposed to formal curriculum (coursework and bedside teaching) felt more prepared and rated their end-of-life care education higher.

Other characteristics such as number of students, medical school location and year of foundation were also associated with MS opinions on ethical issues. Few studies have assessed this relationship, particularly in medical ethics. There are some studies dealing with number of MS and class sizes with some contradictory results such as changes in students’ satisfaction and test grades [25]. In a field such as medical ethics, which requires a closer contact with the teacher, we believe smaller classes could impact the interest and knowledge of the student.

Interestingly, the year of medical school foundation was also associated with MS opinions on controversial ethical issues, in which MS from newer institutions were more supportive to euthanasia, abortion for congenital reasons and embryonic stem cell use. We can speculate that newer medical schools have a less traditional staff than older institutions. In addition, in public institutions, MS were also more supportive, which could be justified by the secular characteristic of these schools compared to private schools, which could have religious affiliations.

Finally, location of the medical school may also impact MS ethical opinions. Rural institutions usually are more conservative and may be less permissive to these controversial ethical issues. Steinauer et al. [26] investigated US obstetrician-gynecologists and found those practicing in rural settings were less likely to provide abortion in clinical practice.

In fact, giving the example of the Brazilian scenario, we have some important differences between types of universities. For instance, in the latest “QS University Rankings: Latin America (2014)”, there are 10 Brazilian universities in the top 20 (9 public universities and 1 private; 7 urban and 3 rural). Likewise, in a recent study in Brazil, Terra et al. [27] found that the public universities have more full-time professors, PhD holders and professors with more years of teaching, and with less religious affiliations compared to private institutions. These remarkable differences could partially explain some of our findings.

To sum up, our results reveal the importance of medical schools characteristics on MS opinions and give further evidence to this area of research. More studies are needed to replicate our findings and to examine MS attitudes and practical skills in hypothetical controversial ethical situations.

Our findings have some limitations that should be highlighted. First, the study was carried out in Brazil and data should be replicated in other cultural contexts. Second, the response rate was 61% and does not include the opinions of over one-third of students who may have had less interest in the topic and so refused to participate. However, this response rate is similar to or better than other multicenter studies in medical education. Third, despite each medical school has a different educational strategy on how to teach medical ethics, we did not evaluate the impact of these strategies on medical students’ ethical opinions.

The present study also has a number of strengths. This is one of the most comprehensive studies of the attitudes and experiences of medical students and, to our knowledge, one of the largest studies dealing with ethics in medical education.

## Conclusions

In conclusion, the current study reveals MS have different opinions regarding controversial ethical issues. Noteworthy, these opinions seem to be shaped more by university characteristics and religious beliefs than socio-demographic data.
